# Lactobacilli Dominance and Vaginal pH: Why Is the Human Vaginal Microbiome Unique?

**DOI:** 10.3389/fmicb.2016.01936

**Published:** 2016-12-08

**Authors:** Elizabeth A. Miller, DeAnna E. Beasley, Robert R. Dunn, Elizabeth A. Archie

**Affiliations:** ^1^Department of Biological Sciences, University of Notre DameNotre Dame, IN, USA; ^2^Department of Biology, Geology and Environmental Science, University of Tennessee at ChattanoogaChattanooga, TN, USA; ^3^Department of Applied Ecology, North Carolina State UniversityRaleigh, NC, USA; ^4^Center for Macroecology, Evolution and Climate, Natural History Museum of Denmark, University of CopenhagenCopenhagen, Denmark; ^5^Institute of Primate Research, National Museums of KenyaNairobi, Kenya

**Keywords:** vaginal microbiome, lactobacilli, pH, estrogen, mammals, evolution

## Abstract

The human vaginal microbiome is dominated by bacteria from the genus *Lactobacillus*, which create an acidic environment thought to protect women against sexually transmitted pathogens and opportunistic infections. Strikingly, lactobacilli dominance appears to be unique to humans; while the relative abundance of lactobacilli in the human vagina is typically >70%, in other mammals lactobacilli rarely comprise more than 1% of vaginal microbiota. Several hypotheses have been proposed to explain humans' unique vaginal microbiota, including humans' distinct reproductive physiology, high risk of STDs, and high risk of microbial complications linked to pregnancy and birth. Here, we test these hypotheses using comparative data on vaginal pH and the relative abundance of lactobacilli in 26 mammalian species and 50 studies (*N* = 21 mammals for pH and 14 mammals for lactobacilli relative abundance). We found that non-human mammals, like humans, exhibit the lowest vaginal pH during the period of highest estrogen. However, the vaginal pH of non-human mammals is never as low as is typical for humans (median vaginal pH in humans = 4.5; range of pH across all 21 non-human mammals = 5.4–7.8). Contrary to disease and obstetric risk hypotheses, we found no significant relationship between vaginal pH or lactobacilli relative abundance and multiple metrics of STD or birth injury risk (*P*-values ranged from 0.13 to 0.99). Given the lack of evidence for these hypotheses, we discuss two alternative explanations: the common function hypothesis and a novel hypothesis related to the diet of agricultural humans. Specifically, with regard to diet we propose that high levels of starch in human diets have led to increased levels of glycogen in the vaginal tract, which, in turn, promotes the proliferation of lactobacilli. If true, human diet may have paved the way for a novel, protective microbiome in human vaginal tracts. Overall, our results highlight the need for continuing research on non-human vaginal microbial communities and the importance of investigating both the physiological mechanisms and the broad evolutionary processes underlying human lactobacilli dominance.

## Introduction

Comparative research on mammalian microbiomes is critical to understanding the basic evolutionary and ecological principles guiding microbiome structure and function across mammalian hosts (Ley et al., 2008b; Delsuc et al., 2014; Yildirim et al., 2014; Moeller et al., 2016). To date, most comparative studies in mammals find that hosts with similar lifestyles and evolutionary histories harbor similar microbiomes at a given body site, both in the bacterial taxa they contain and the functions they provide to hosts (Ley et al., 2008a; Delsuc et al., 2014). One important exception to this pattern is the vaginal microbiome, where humans exhibit striking differences in community composition compared to other mammals (Spear et al., 2012; Swartz et al., 2014; Yildirim et al., 2014). Specifically, the human vaginal microbiome is dominated by *Lactobacillus* spp., which typically comprise >70% of resident bacteria in women, compared to <1% in other mammals. These lactobacilli process glycogen and its breakdown products to produce lactic acid, leading to an exceptionally low vaginal pH of ≤4.5 (Boskey et al., 1999, 2001; Mirmonsef et al., 2014; Spear et al., 2014). Lactobacilli-dominance and low pH of the human vaginal microbiome are hypothesized to benefit women by reducing disease risk (reviewed in Brotman, 2011; Graver and Wade, 2011; O'Hanlon et al., 2011; Gong et al., 2014; Nunn et al., 2015). Furthermore, the loss of lactobacilli-dominance is linked to bacterial vaginosis (BV), which is associated with an overgrowth of anaerobic bacteria, relatively high vaginal pH (>4.5; Aldunate et al., 2015), infertility, preterm birth, maternal infections, and increased risk of sexually transmitted diseases (Cherpes et al., 2003; Leitich et al., 2003; Atashili et al., 2008; Brotman et al., 2010; van Oostrum et al., 2013; DiGiulio et al., 2015; Redelinghuys et al., 2016). The fact that the human vaginal microbiome appears to be unique among mammals raises key questions about why humans are different: Do humans have a distinct reproductive physiology or experience different or stronger forces of selection compared to other mammals? Or are the selective pressures experienced by humans common among mammals, but humans have found a unique microbial solution to these evolutionary forces?

To date, four non-mutually exclusive hypotheses have been proposed to explain the uniqueness of the human vaginal microbiome relative to other primates, and, by extension, to other mammals (Stumpf et al., 2013). Two of these hypotheses focus on proximate, mechanistic explanations, and two propose ultimate or evolutionary explanations. The first proximate explanation, the “reproductive phase hypothesis,” proposes that the differences between human and non-human mammal vaginal microbiomes are due to between-species differences in reproductive physiology—especially the fact that humans exhibit continuous ovarian cycling, while many other mammals do not. Humans experience continuous, 28-day ovarian cycles between menarche and menopause, governed by fluctuations in reproductive steroids. In humans, estrogen levels are closely linked to lactobacilli abundance and vaginal pH, with an increase in estrogen promoting the thickening of the vaginal epithelium and intracellular production of glycogen (Ayre, 1951; Nauth and Haas, 1985; Patton et al., 2000; but see Mirmonsef et al., 2016). Consequently, lactobacilli are most abundant and vaginal pH is lowest when estrogen levels peak just before ovulation (Drake et al., 1980; Wagner and Ottesen, 1982; Eschenbach et al., 2000). In many non-human primates, and indeed in many mammals, females do not cycle continuously and are often only sexually receptive during a distinct breeding season (Hayssen et al., 1993; Noakes et al., 2009; Dixson, 2012). While estrogen levels follow similar patterns during the ovarian cycles of non-human mammals (Noakes et al., 2009; Dixson, 2012), many species may only experience high estrogen, high lactobacilli abundance, and low vaginal pH during a brief period of time that is commonly undetected by researchers (Stumpf et al., 2013). Thus, the reproductive phase hypothesis predicts that human uniqueness may be, in part, an artifact of sampling other mammals at the wrong time—outside of high estrogen cycle phases.

The second proximate explanation, called “the common function hypothesis,” proposes that, in non-human mammals, other bacteria may protect hosts via mechanisms other than lactic acid and low vaginal pH, such as production of bacteriocins and other antimicrobial compounds, competitive exclusion, or interactions with the host immune system (Klaenhammer, 1988; Abt and Artis, 2013; Stumpf et al., 2013; Aldunate et al., 2015). Thus, the presence of lactobacilli may not be a requirement for a healthy vaginal environment. This explanation may also be relevant to human health as some women consistently have low abundance of lactobacilli and a vaginal pH > 4.5, but they do not experience negative symptoms associated with BV (Ravel et al., 2011).

In addition to these mechanistic explanations, two evolutionary hypotheses have been proposed to explain why the human vaginal microbiome appears to be unique among mammals. The first evolutionary hypothesis, referred to as the “disease risk hypothesis,” proposes that humans face higher sexually transmitted disease (STD) risk than non-human mammals. Species with promiscuous mating strategies are predicted to have higher STD risk than those with only a single, brief reproductive episode per breeding season (Thrall et al., 1997, 2000; Nunn et al., 2000, 2003; Nunn, 2002). Humans, in particular, may experience relatively high STD risk due to prolonged intromission and continuous sexual receptivity throughout the menstrual cycle, pregnancy, and the postpartum period. Since STDs may have a significant impact on host and host offspring fitness (Lockhart et al., 1996), selective pressure for a protective, lactobacilli-dominated community may be stronger in humans compared to mammals with less frequent sexual contact (Stumpf et al., 2013).

Along a similar line of reasoning, the “obstetric protection hypothesis” suggests that selection for lactobacilli in the human vagina is due to the high risk of microbial complications associated with pregnancy and childbirth. Problems associated with gestation and parturition are not uncommon in mammals (e.g., Aksel and Abee, 1983; Sheldon et al., 2006), but humans may experience particularly difficult pregnancies and births (Rosenberg and Trevathan, 2002). For example, since the human maternal pelvic outlet is smaller than the neonatal head, there is substantial risk of trauma to uterine and vaginal walls, which increases the likelihood of microbial infection (Rosenberg and Trevathan, 2002; Chaim and Burstein, 2003; Dajani and Magann, 2014). Thus, lactobacilli and low vaginal pH may serve a protective function during human birth, and these traits are unnecessary in mammals with less risky pregnancies and birth (Stumpf et al., 2013).

Our objective was to test these hypotheses by comparing vaginal microbiomes and vaginal pH across mammalian species with a range of reproductive physiologies, mating systems, and obstetric risks. Recently, a comparative analysis of the vaginal microbiome across non-human primates found that interspecies differences were predominantly explained by host species, with additional evidence that host socio-ecological factors, including host neonatal birth weight, may contribute to interspecies variation (Yildirim et al., 2014). However, no studies have yet directly correlated specific host risk factors with lactobacilli abundance and vaginal pH, the aspects of the vaginal microbiome thought to be protective in humans. Furthermore, comparative data on vaginal microbial composition—especially interspecies and inter-individual variation in the vaginal microbiome as a function of female reproductive state and ovarian cycle phase—are rare. Here, we test three of these hypotheses—the reproductive phase, disease risk, and obstetric protection hypotheses—using comparative data on vaginal pH from 21 species of mammals and lactobacilli relative abundance from 14 species of mammals, as a function of female reproductive physiology, STD risk, and obstetric risk. To test the reproductive phase hypothesis, we predicted that: (i) non-human mammals would exhibit the lowest vaginal pH during the phase of their cycle with the highest estrogen, and (ii) at peak estrogen, non-human vaginal pH would be indistinguishable from humans. For the disease risk hypothesis, we predicted that species with high STD risk would possess lower vaginal pH and higher lactobacilli relative abundance than those species with low STD risk. Similarly, for the obstetric protection hypothesis, we predicted that mammals with high obstetric risk would have lower vaginal pH and higher lactobacilli relative abundance than species with low obstetric risk. Overall, this work represents one of the first attempts to explain interspecies variation in the vaginal microbiome, particularly with regard to human uniqueness (but see Yildirim et al., 2014).

## Materials and methods

### Literature search and inclusion criteria

**Figure 1:**
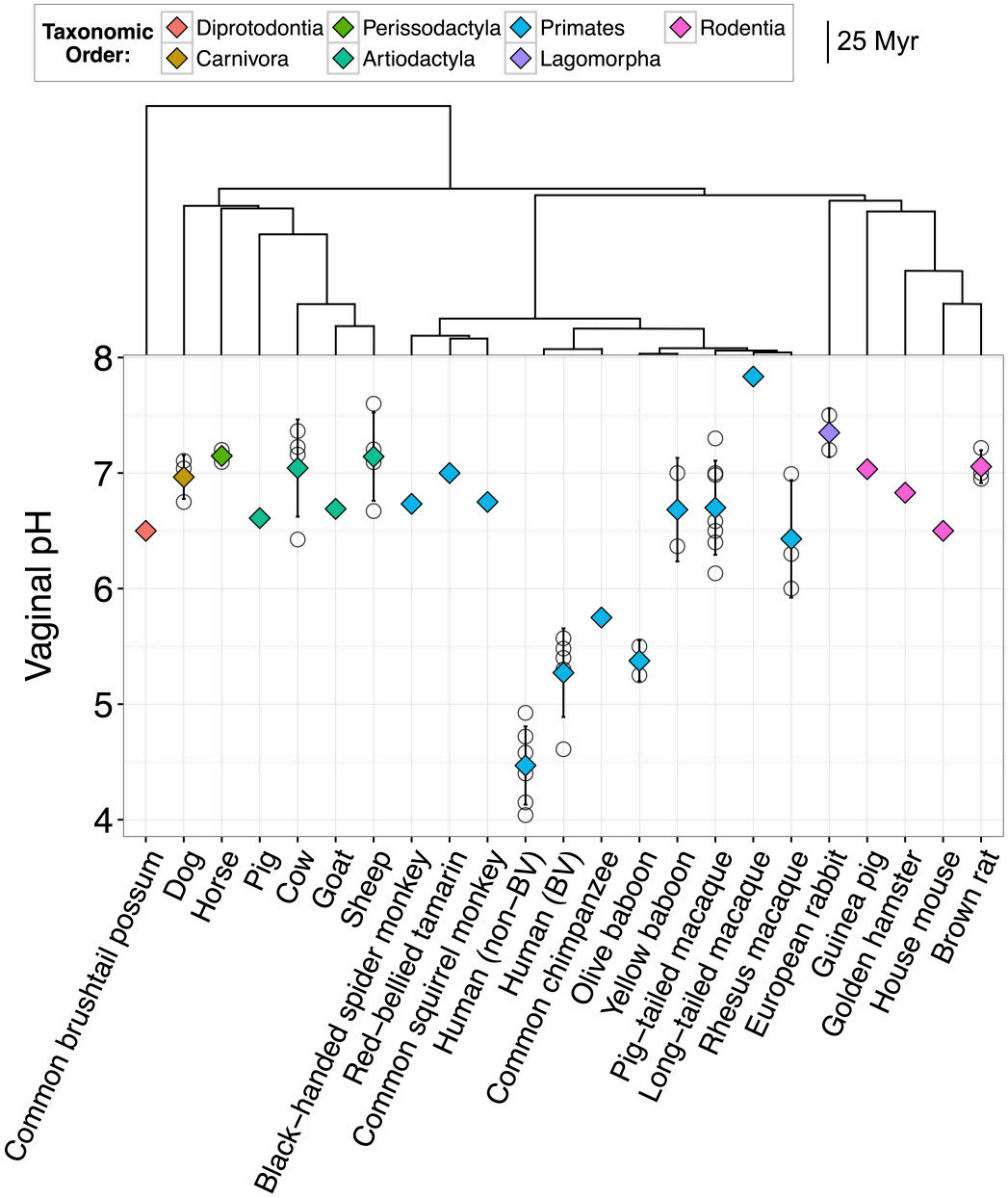
**Vaginal pH across 22 species of mammals including humans**. Open circles represent mean pH from individual studies and diamonds represent the overall mean for that species. Diamonds are color-coded based on taxonomic order. Error bars represent the standard deviation from the mean. Humans are divided into two groups, one with bacterial vaginosis (BV) and one without BV. Dendrogram indicates the evolutionary distance between species in millions of years (Myr).

We searched for data on mammalian vaginal lactobacilli and pH using appropriate search terms in Web of Science, Google Scholar, PubMed, and in the references of other publications. For information on lactobacilli prevalence and relative abundance, we focused on culture-independent studies (i.e., lactobacilli identification based on Illumina or 454 sequencing) because cultivation-based methods can under-represent or overlook some species (Zhou et al., 2004; Lamont et al., 2011). For data on vaginal pH, all pH measurements were collected using standard methods with pH meters or paper. Of particular note, human pH measurements from O'Hanlon et al. (2013) were not included because they collected data under hypoxic conditions and therefore were not comparable to measurements on non-human mammals. In addition, because the human vaginal microbiome changes in response to puberty and menopause and between reproductive states (e.g., Cauci et al., 2002; Thoma et al., 2011a; MacIntyre et al., 2015), we confined our data set to pH values collected during the ovarian cycle (i.e., follicular and luteal phases, anestrus) of sexually mature subjects. Furthermore, humans or non-human animals who had recently mated were not included as semen is alkaline and can temporarily neutralize vaginal tract pH (Tevi-Bénissan et al., 1997). Finally, because testing the reproductive phase hypothesis required data on how natural fluctuations in estrogen impact vaginal pH, we excluded subjects who were sterilized or supplemented with exogenous hormones. However, we did include studies of humans where women were taking hormonal birth control because past work has found no relationship between vaginal pH and birth control (Drake et al., 1980; Wagner and Ottesen, 1982). In total, we found 50 studies with data on vaginal pH and/or lactobacilli relative abundance across 26 mammalian species. Specifically, we found information on vaginal pH for 21 different species of non-human mammals from 44 studies (Figure 1; Table S1) and data on relative abundance of lactobacilli for 14 mammals, 8 of which also had information on vaginal pH (Table 2).

### Calculating vaginal pH

For each mammalian species, we calculated the average vaginal pH across all available studies (Table S1). Because some studies measured vaginal pH at only a few cycle phases, the average pH of a species reflects the available data and not necessarily all cycle phases. If a study reported a pH range instead of a single value, we calculated the midrange (i.e., added the minimum and maximum values and divided by 2). For studies that represented their data graphically, but did not give numerical values, we used *WebPlotDigitizer* (Rohatgi, 2015) to extract pH values from the relevant plots. To test the reproductive phase hypothesis, we used vaginal pH values from the phases of the ovarian cycle when estrogen was highest and lowest (Table S1). For the majority of mammalian species, peak estrogen occurs during late proestrus or estrus, and the lowest level of estrogen occurs during the luteal phase or menses (Figure S1). However, for several species, including cows and dogs, peak estrogen occurs at both the end of proestrus and beginning of estrus. In these instances, we used the pH value from estrus to represent the high estrogen time point because proestrus can span multiple days and can encompass considerable variation in estrogen levels (Figure S1).

### Measuring STD and obstetric risk

To test the disease risk and obstetric protection hypotheses, we required information on interspecies risk associated with STDs and reproduction. We calculated proxies of both STD and obstetric risk using species-specific trait data compiled from primary literature, edited books, and the life history database, PanTHERIA (Jones et al., 2009; Table S2). Whenever possible, we used data from wild, feral, or free-ranging populations over data from captive or domesticated individuals. In 13 instances, we could not find a particular life history trait for a species. In these cases, we used data from the closest related species with available data. For example, there was no information on the age of sexual maturity or life expectancy for the red-bellied tamarin (*Sanginus labiatus*), so we used data from the closely related cotton-top tamarin (*S. oedipus*). All proxies and their descriptions are listed in Table 1. Specifically, to test the disease risk hypothesis, we used five proxies: testes mass relative to body size, baseline white blood cell (WBC) count, annual sexual receptivity, total lifetime reproductive events, and intromission pattern (Table 1). For the obstetric protection hypothesis, we used gestation length, relative neonatal mass, and relative maternal pelvic area (Table 1).

**Table 1:** **Proxies of mammalian STD and obstetric risk**.

**Proxy**	**Description**
**STD RISK**	
(1) Relative testes mass	After correcting for body size, testes mass indicates degree of sperm competition, which gives an estimate of promiscuity between species (Harcourt et al., 1981; Kenagy and Trombulak, 1986).
(2) White blood cell count	Species with high risk of STDs should have a corresponding high number of baseline WBCs in order to cope with potential infections (Nunn et al., 2000, 2003; Nunn, 2002).
(3) Annual sexual receptivity	Species that experience longer periods of sexual receptivity may experience increased STD risk (van Schaik et al., 1999). To correct for variability in cycle length across mammals, we standardized sexual receptivity duration to 1 year:
	$sexual\;receptivity\;\left(days\right)*\;\frac{annual\;breeding\;season\;\left(days\right)}{ovarian\;cycle\;\left(days\right)}$
(4) Total lifetime reproductive events	Species with longer lifespans and more reproductive events may experience increased STD risk (Loehle, 1995; Nunn, 2003).
	$\frac{maximum\;lifespan-age\;at\;first\;reproduction}{interbirth\;interval}$
(5) Intromission pattern	During copulation, more intromissions and longer intromission duration increase the probability of STD exposure and transmission. Following the categories in Dixson (2012), we classified the intromission pattern of a species as Single or Multiple and Brief (≤3 min) or Prolonged (>3 min). Thus, a species was assigned one of four overall intromission patterns: MBI, SBI, MPI, or SPI.
**OBSTETRIC RISK**	
(6) Gestation length	Gestation can be an energetically costly reproductive event and may increase maternal susceptibility to certain types of infections via changes in the immune system (Gittleman and Thompson, 1988; Krishnan et al., 2013). Additionally, physiological changes that occur during gestation may disrupt maternal microbial communities (Redelinghuys et al., 2016).
(7) Relative neonatal mass	In humans, larger neonates increase the likelihood of complications during birth (Alsammani and Ahmed, 2012; Weissmann-Brenner et al., 2012). From the interspecies perspective, species with larger neonatal mass, controlling for maternal body mass, may experience more risk associated with parturition than species with relatively small neonates (Leutenegger, 1973).
(8) Relative maternal pelvic area	Species with small maternal pelvic inlet areas relative to neonatal mass may be more prone to complications in passing neonates through the maternal birth canal (Deutscher, 1985; Basarab et al., 1993; Rosenberg and Trevathan, 2002). We calculated this proxy as:
	$\frac{maternal\;pelvic\;inlet\;area}{neonatal\;mass}$
	Note: Because pelvic inlet measurements were not available for many species in our dataset, this proxy could only be calculated for a subset of species.

### Hypothesis testing

All statistical tests were implemented in the R statistical environment (R Core Team, 2015). Before testing our primary hypotheses, we first tested for a phylogenetic signature on vaginal pH such that mammals with more similar evolutionary histories had more similar vaginal pH values. We obtained divergence times between the species in our data set from Bininda-Emonds et al. (2007), with an updated primate phylogeny by Perelman et al. (2011) and *Papio* spp. values from Purvis (1995). We constructed a distance matrix and phylogenetic tree using the R package, *ape* (Paradis et al., 2004), along with a distance matrix of vaginal pH values between species. We correlated phylogenetic distance with differences in vaginal pH using a Mantel test, which calculates a *P*-value based on comparison of the observed Pearson correlation coefficient to the distribution of coefficients from 10,000 permutations.

To test the reproductive phase hypothesis, we compared the vaginal pH during high- and low-estrogen phases of the female ovarian cycle using a paired *t*-test in R. To test the disease risk and obstetric protection hypotheses, we correlated vaginal pH and lactobacilli relative abundance with each proxy of STD or obstetric risk using linear regressions and ANOVAs. To calculate the minimum sample size required to detect a significant relationship, we used the R package, *pwr* (Champely, 2015), with a power value of 0.80. We further constructed multivariate linear regression models to test whether any combination of proxies predicted vaginal pH. Predictor variables in each model included relative testes mass, baseline WBC count, annual sexual receptivity, total lifetime reproductive events, intromission pattern, gestation length, and relative neonatal mass. Model selection was performed using stepwise backward regression with the *stepAIC* function from the R package, *MASS* (Venables and Ripley, 2002) and a 5% significance level was used as a threshold for inclusion in the final model.

## Results and discussion

### Interspecies comparison of lactobacilli and vaginal pH reflects the unique nature of the human vaginal microbiome

Of the 26 mammalian species for which we found data on vaginal pH and/or lactobacilli relative abundance, most were captive primates used for medical studies, domesticated ungulates (e.g., horse, cow, pig), and common laboratory rodents (e.g., mouse, rat, and guinea pig). Of note, only one study measured vaginal pH in a wild population (yellow baboons; Miller et al., in review).

**Figure 2:**
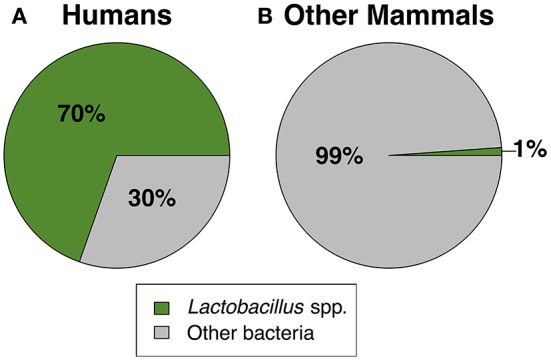
**The mean relative abundance of *Lactobacillus* spp. vs. other bacteria in (A)** humans and **(B)** non-human mammals. For non-human mammals, lactobacilli relative abundance was calculated as the mean across all species (*N* = 14). The standard error of the mean for lactobacilli was ±0.046% in humans and ±0.39% in other mammals. See Table 2 for the list of studies used to generate this figure.

Together, our results confirm that humans are distinct from other mammals in dominance of lactobacilli and the acidity of their vaginal tract. For instance, across 10 studies of healthy human women, median vaginal pH was 4.5 (range = 4.0–4.9), while median vaginal pH across non-human mammals was 6.8, with no species falling in the range of healthy human pH, and only two species with a pH below 6.0 (Figure 1). Furthermore, while 13 of 14 mammals had detectable levels of *Lactobacillus* spp., the average relative abundance of lactobacilli was only 1.1% (±0.39% SEM) in non-human mammals compared to 69.6% in human women (±0.046% SEM) (Figure 2). This disparity is unlikely to be due to differences in the species of *Lactobacillus* in non-human mammals as compared to humans as the same four species that dominate the vaginal tract of women (*L. cripsatus, L. gasseri, L. iners*, and *L. jensenii*), were also frequently found in other mammals, albeit in low relative abundance. However, non-human mammals also harbored other lactobacilli generally not found in humans, including *L. animalis, L. fornicalis, L. amylovorus*, and *L. johnsonii*. Other members of the phylum Firmicutes were also common in non-human mammals, particularly species of two lactic acid-producing genera *Aerococcus* and *Facklamia*. Among primates, there was also high relative abundance of multiple genera linked to bacterial vaginosis (BV) in women, including *Gardnerella, Sneathia*, and *Prevotella* (Onderdonk et al., 2016).

**Table 2:** **Prevalence and relative abundance of the genus *Lactobacillus* across mammalian species**.

**Order**	**Species**	**Common name**	**Origin^a^**	***N***	**Lactobacilli prevalence (%)**	**Lactobacilli relative abundance (%)**		**References**
						**Mean ± SD**	**Range**	
**RODENTIA**								
	*Cavia porcellus*	Guinea Pig	D	5	100	0.013±0.0079%	0.0017–0.021%	Neuendorf et al., 2015
**ARTIODACTYLA**								
	*Bos taurus*	Cow	D	20	90	0.36±0.66%	–	Swartz et al., 2014
	*Ovis aries*	Sheep	D	20	80	0.53±0.65%	–	Swartz et al., 2014
	*Sus scrofa*	Pig	D	20	≥90	3.52±0.45%	3.00–4.20%	Lorenzen et al., 2015
**PRIMATES**								
	*Alouatta pigra*	Black Howler	W	5	0	−	–	Yildirim et al., 2014
	*Cercocebus atys*	Sooty Mangabey	C	6	100	4.77±7.56%	0.12–19.69%	Yildirim et al., 2014
	*Chlorocebus aethiops*	Grivet	C	6	100	1.68±3.16%	0.026–8.05%	Yildirim et al., 2014
			W	6	83.33	1.42±3.11%	0–7.77%	Yildirim et al., 2014
	*Homo sapiens*	Human	–	9	100	77±29.47%	2.99–97.99%	Yildirim et al., 2014
			–	398	98.50	70.62±39.4%	0–99.85%	Ravel et al., 2011
			–	32	100	61.21±35.65%	0.020–99.65%	Gajer et al., 2012
	*Macaca mulatta*	Rhesus Macaque	C	11	36	generally <1%	0–39%	Spear et al., 2010
	*Macaca nemestrina*	Pig-Tailed Macaque	C	10	100	2.20%	<1–27%	Spear et al., 2012
	*Pan troglodytes*	Common Chimpanzee	W	12	75	0.33±0.91%	0–3.21%	Yildirim et al., 2014
	*Papio anubis*	Olive Baboon	C	3	100	generally <1%	<1–9%	Hashway et al., 2014
			C	38	16	1.25±3.34	0–14.95%	Uchihashi et al., 2015
			C	6	100	2.09%±2.6%	0.12–7.20%	Yildirim et al., 2014
	*Papio cynocephalus*	Yellow Baboon	W	48	84.6	0.036±0.14%	0–0.93%	Miller et al., in review
			W	6	33.33	0.014±0.028%	0–0.071%	Yildirim et al., 2014
	*Procolobus rufomitratus*	Red Colobus	W	6	33.33	0.021±0.035%	0–0.084%	Yildirim et al., 2014
	*Propithecus diadema*	Diademed Sifaka	W	6	33.33	0.0022±0.0035%	0–0.0080%	Yildirim et al., 2014

*All data are from culture-independent studies (i.e., Illumina and 454 sequencing)*.

a *Captive (C), Wild (W), or Domesticated (D)*.

Importantly, as has been reported previously, human women with BV had significantly higher vaginal pH than healthy women (*N* = 6 studies; two-sample *t*-test: *t*_(8.14)_ = 3.65, *P* = 0.0063; Figure 1). Women with BV also exhibited compositional similarities to healthy baboons and macaques, including moderately high relative abundances of *Gardnerella, Mobiluncus, Sneathia*, and *Prevotella* (e.g., Miller et al., in review; Spear et al., 2010, 2012; Uchihashi et al., 2015; Onderdonk et al., 2016). However, of the five macaque and baboon species in our data set, all but the olive baboon had significantly higher vaginal pH than BV women (ANOVA: *F*_(5, 14)_ = 12.84, *P* = 8.12e-05; Figure 1), which suggests that similar microbial composition may not always translate into similar ecological functions and consequences (e.g., Mirmonsef et al., 2012).

### Phylogeny does not predict vaginal pH

Before testing our main hypotheses, we first investigated whether shared evolutionary history among mammals explained variation in vaginal pH among mammal species. Figure 1 indicates that primates exhibited the widest variation in vaginal pH, with species-specific averages in non-human primates ranging from 5.4 to 7.8, and chimpanzees and olive baboons exhibiting pH values most similar to humans. Indeed, more variation in vaginal pH existed among primate species than among all other mammal lineages, which, despite encompassing many more years (and units of branch length) of evolutionary history, had vaginal pH values that ranged from just 6.5–7.4 (Figure 1). However, evolutionary distance between mammalian species did not predict similarity in vaginal pH, either among humans and non-human primates (Mantel test: *N* = 10, Mantel statistic *r* = −0.01, *P* = 0.47), or among all mammals (*N* = 22, Mantel statistic *r* = −0.2, *P* = 0.93). Hence, shared evolutionary history is unlikely to play a dominant role in shaping interspecies variation in vaginal pH.

### Across mammals, females exhibit low vaginal pH during periods of peak estrogen

**Figure 3:**
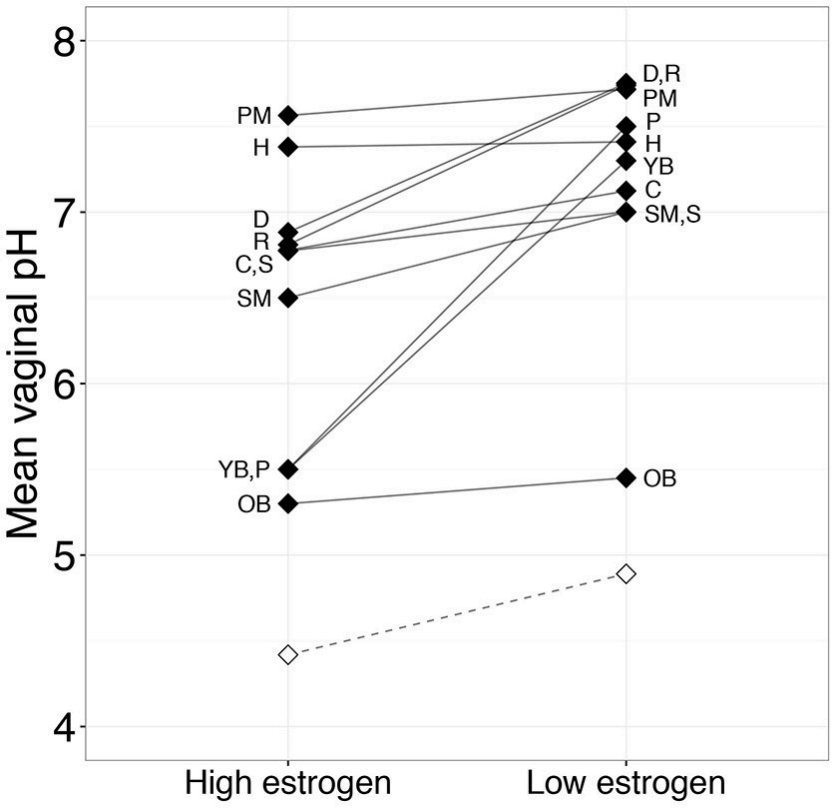
**Mean vaginal pH between periods of high and low estrogen of 11 mammalian species during the ovarian cycle**. Paired black diamonds represent the overall mean for each species at both estrogen levels. The open diamonds and dashed line show the mean human vaginal pHs during the high estrogen phases (follicular phase and ovulation) and low estrogen phases (luteal phase and menstruation) of the ovarian cycle. Letters next to diamonds identify the mammalian species. See Table S1 for the list of studies used to generate this figure. Abbreviations: C, Cow; D, Dog; H, Horse; OB, Olive baboon; P, Common brushtail possum; PM, Pig-tailed macaque; R, Brown rat; S, Sheep; SM, Black-handed spider monkey; YB, Yellow baboon.

The reproductive phase hypothesis proposes that differences between human and non-human primate vaginal microbiota are due to between-species variation in reproductive physiology—especially the continuous nature of human ovarian cycling. This hypothesis predicts that non-human mammals will exhibit lactobacilli dominance and a vaginal pH similar to humans during the period in their ovarian cycle when they experience highest estrogen levels. To test this idea, we compared vaginal pH during high- and low-estrogen phases of the female ovarian cycle in 10 non-human mammals where we had enough information on pH throughout the ovarian cycle. Notably, some of the mammal species we considered exhibit continuous, year-round reproductive cycling (i.e., spider monkey, cow, rat, pig-tailed macaque, common brushtail possum, and olive and yellow baboons), allowing us to make a strong comparison with humans. We found that, similar to humans, non-human mammals exhibit the lowest vaginal pH during high-estrogen phases (paired *t*-test: *t*_(9.00)_ = −3.16, *P* = 0.012; Figure 3). However, even during peak estrogen, the pH of non-human mammals never declined to a level comparable to humans (Figure 3). Data on the relative abundance of *Lactobacillus* spp. as a function of ovarian cycle phase are rare in non-human mammals, but one study on wild baboons found that the order Lactobacillales, which comprises all lactic acid-producing bacteria, including lactobacilli, was more abundant in individuals experiencing ovulation compared to other ovarian cycle phases (Miller et al., in review). However, the mean relative abundance of *Lactobacillus* spp. in these ovulating baboons was only 0.0058%, which is considerably lower than the level typical in healthy human women (Miller et al., in review).

Together, these results suggest that estrogen plays a similar role in shaping vaginal microbial composition and pH in humans and in other mammals. Specifically, rising estrogen levels increase available glycogen in the vaginal epithelium, which in turn, provides an energy source for lactobacilli to produce lactic acid. Indeed, like humans, many non-human mammals exhibit a thickening of the vaginal epithelium and increasing glycogen content in response to rising estrogen levels during the ovarian cycle (e.g., Gregoire and Guinness, 1968; Gregoire and Parakkal, 1972; Williams et al., 1992; Nyachieo et al., 2009). However, direct correlations between estrogen, glycogen, lactic acid, lactobacilli, and vaginal pH have yet to be explored in non-human mammals to the extent they have been in humans (e.g., Boskey et al., 1999, 2001; Mirmonsef et al., 2014; but see Mirmonsef et al., 2012). In summary, in terms of the reproductive phase hypothesis, we find that differences in reproductive cycling between humans and other mammals are not sufficient to explain why the human vaginal pH and lactobacilli abundance are such outliers relative to other mammals. Hence the uniqueness of the human vaginal microbiome is unlikely to be a consequence of sampling non-human mammals during the incorrect reproductive state.

### Vaginal pH and lactobacilli relative abundance across mammals does not correlate with risk associated with sexually transmitted diseases or obstetrics

**Figure 4:**
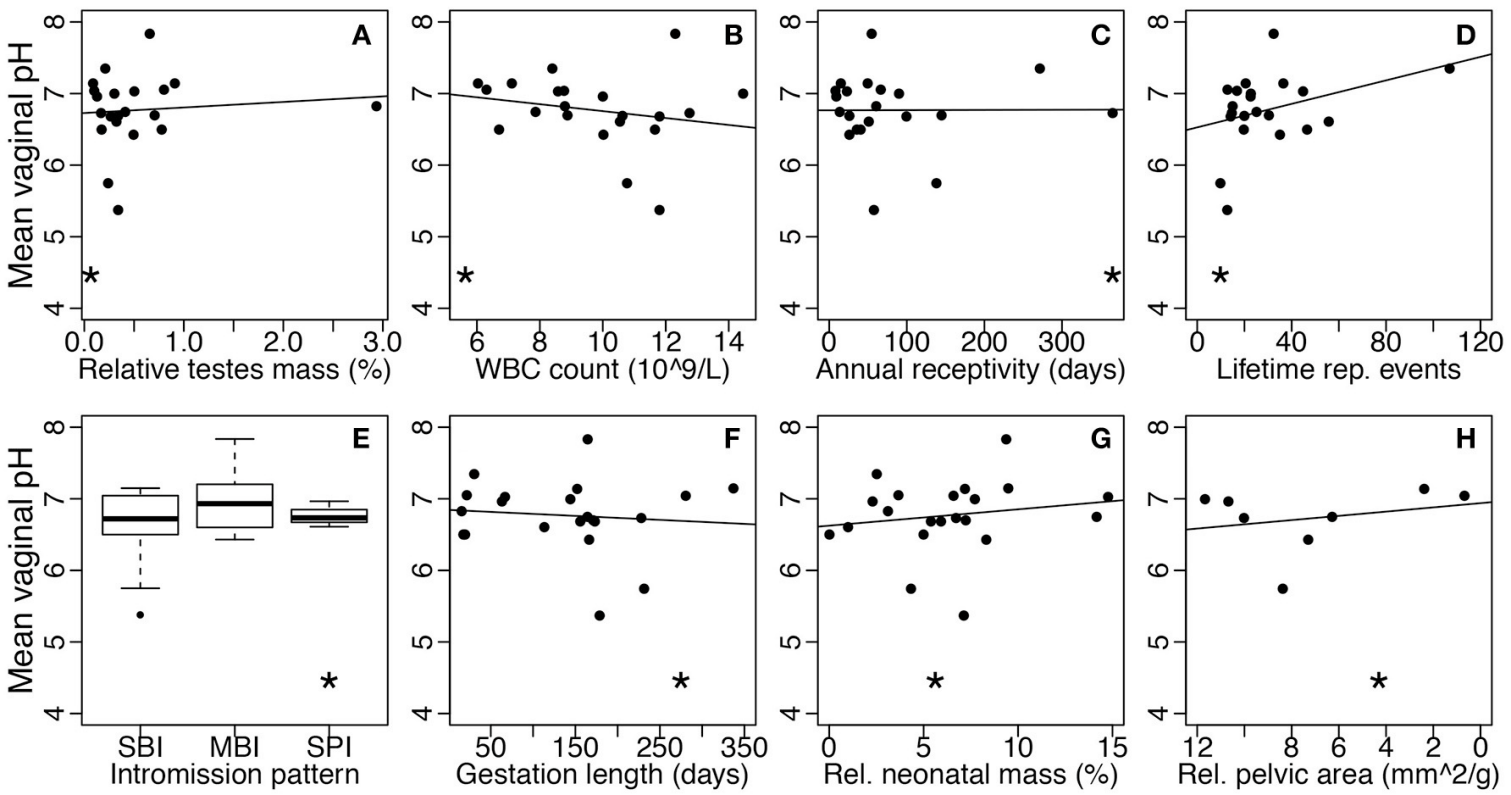
**Mean vaginal pH as a function of STD or obstetric risk across mammals**. Each point represents one species. Asterisks show where humans fall within each comparison. STD risk proxies are **(A)** relative testes mass, **(B)** baseline white blood cell count, **(C)** annual sexual receptivity, **(D)** maximum lifetime reproductive events, and **(E)** intromission pattern (SBI, single brief intromission; MBI, multiple brief intromissions; SPI, single prolonged intromission). Obstetric risk proxies are **(F)** gestation length, **(G)** relative neonatal mass, and **(H)** relative maternal pelvic area. Risk level increases moving left to right on all plots (note the reversed x-axis in plot **H**). The solid lines represent the best-fit linear models without humans.

The disease risk hypothesis proposes that high risk of STD exposure in humans, compared to other mammals, has selected for a protective, lactobacilli-dominated community. This hypothesis predicts that, in general, mammals, such as humans, with greater STD risk will have higher abundance of lactobacilli and lower vaginal pH compared to species with minimal STD risk. To test this hypothesis, we correlated the vaginal pH and lactobacilli relative abundance of mammals with species-specific proxies of STD risk (Table 1). Overall, we found no significant relationships between vaginal pH and any of the five STD risk proxies, including relative testes mass (linear regression: *N* = 21, *F*_(1, 19)_ = 0.16, *P* = 0.70; Figure 4A), baseline WBC count (*N* = 21, *F*_(1, 19)_ = 0.90, *P* = 0.36; Figure 4B), annual sexual receptivity (*N* = 21, *F*_(1, 19)_ = 0.00030, *P* = 0.99; Figure 4C), number of lifetime reproductive events (*N* = 21, *F*_(1, 19)_ = 2.56, *P* = 0.13; Figure 4D), and intromission pattern (ANOVA: *N* = 21, *F*_(2, 18)_ = 1.07, *P* = 0.36; Figure 4E). Additionally, there was no significant relationship between lactobacilli relative abundance and any of the same STD risk proxies (*N* = 14; relative testes mass: *F*_(1, 12)_ = 0.0050, *P* = 0.95; baseline WBC count: *F*_(1, 12)_= 0.072, *P* = 0.79; annual sexual receptivity: *F*_(1, 12)_ = 0.79, *P* = 0.39; number of lifetime reproductive events: *F*_(1, 12)_ = 2.66, *P* = 0.13; copulation pattern: *F*_(2, 11)_ = 2.52 *P* = 0.13).

Parallel to the disease risk hypothesis, the obstetric protection hypothesis proposes that the unique nature of the human vaginal microbiome is due to risk of infection during human pregnancy and birth. By extension, across mammals, species experiencing higher risk during gestation and parturition should have higher abundance of lactobacilli and lower vaginal pH. We correlated vaginal pH and lactobacilli relative abundance with three common proxies of obstetric risk across mammals (Table 1). Again, there was no relationship between vaginal pH and any obstetric risk proxy, including gestation length (linear regression: *N* = 21, *F*_(1, 19)_ = 0.18, *P* = 0.67; Figure 4F), relative neonatal mass (*N* = 21, *F*_(1, 19)_ = 0.54, *P* = 0.47; Figure 4G), and relative maternal pelvic area (*N* = 11, *F*_(1, 6)_ = 0.42, *P* = 0.54; Figure 4H). There was also no relationship between lactobacilli relative abundance and any proxy (gestation length: *N* = 14, *F*_(1, 12)_ = 0.69, *P* = 0.42; relative neonatal mass: *N* = 14, *F*_(1, 12)_ = 0.20, *P* = 0.66; relative maternal pelvic area: *N* = 5, *F*_(1, 3)_ = 0.0054, *P* = 0.95).

Finally, because disease and obstetric risk could be acting as simultaneous selection pressures on the vaginal microbiome, we also tested both hypotheses together using combinations of STD and obstetric risk proxies as factors in multivariate linear regressions predicting vaginal pH and lactobacilli relative abundance. Consistent with univariate results, no combination of proxies was significantly correlated with either vaginal pH or lactobacilli relative abundance (*P*-value range = 0.89–0.13). While the sample sizes for these correlations were small, a power analysis suggests that we still would need a sample size at least 2 times our current sample size and often more than 10 times the current sample size to detect significant effects. Hence, STD and obstetric risk are, at best, minor forces shaping mammalian vaginal pH and the relative abundance of lactobacilli.

Our results suggest that, contrary to the assumptions underlying the disease risk and obstetric protection hypotheses, humans do not have considerably higher disease or obstetric risk compared to other mammals (see asterisks on Figure 4). For example, many mammals have STDs and may experience high STD risk due to sexually promiscuous behavior (Smith and Dobson, 1992; Lockhart et al., 1996; Altizer et al., 2003). Additionally, a number of mammals have longer gestation duration than humans, and may experience extreme complications associated with parturition (Aksel and Abee, 1983; Frank and Glickman, 1994; Rosenberg and Trevathan, 2002). For instance, squirrel monkeys give birth to exceptionally large neonates for the diameter of the maternal pelvis, which can lead to an almost 50% perinatal mortality in some captive populations, yet vaginal pH in squirrel monkeys is around 6.75 (Aksel and Abee, 1983). Thus, the human STD and obstetric risk selective pressures do not appear to be particularly strong compared to selective pressures experienced by other mammals. Together, these results do not support the hypotheses that STD or obstetric risks have shaped the mammalian vaginal microbiome.

### Alternative hypotheses

#### The common function hypothesis

While we found little support for three of the four hypotheses proposed to explain the unique nature of the human vaginal microbiome (Stumpf et al., 2013), the fourth hypothesis (i.e., the common function hypothesis) could not be tested with available data. This hypothesis remains a promising avenue for future work. Specifically, it suggests that while all mammals may experience similar selective pressures, humans have found a unique microbial solution in lactobacilli-dominance. Further, in other host species, the protective role of lactobacilli may be fulfilled by other microbes that achieve similar functions without low vaginal pH. For example, vaginal bacteria, including some microbes commonly found at high abundances in the vaginal tract of non-human mammals (e.g., *Streptococcus, Prevotella*, and *Corynebacterium*), may produce high quantities of antimicrobial proteins, called bacteriocins (Zheng et al., 2015). Additionally, the microbes of non-human mammals may provide a protective function via interactions with the host immunity. Indeed, in humans, lactobacilli and lactic acid are thought to mediate host immune responses in vaginal mucosa, while BV-associated bacteria tend to promote pro-inflammatory immune responses (Mirmonsef et al., 2011; Aldunate et al., 2015). In non-human mammals, the interaction between commensal vaginal bacteria and host immunity have yet to be extensively investigated and it remains to be seen whether those interactions, particularly with regard to lactic acid and BV-associated bacteria, are similar to what is observed in humans or are species-specific.

#### Glycogen and the human diet

While the common function hypothesis may clarify how humans and other mammals differ in their protective microbial communities, it does not explain why humans possess a unique microbial solution to relatively similar selective pressures. Here we propose a new proximate explanation for the uniqueness of human vaginal microbiome, which posits that exceptionally high levels of glycogen in the human vaginal tract create “lactobacilli-friendly” conditions, leading to lactobacilli dominance. While data on glycogen levels in non-human mammals are rare, the limited data that exist indicate that humans have considerably higher concentrations of glycogen present in vaginal epithelial tissue and in genital fluid compared to other mammals (Table 3; Mirmonsef et al., 2012). Because glycogen and its breakdown products (e.g., maltose) are a main energy source for lactobacilli, low glycogen levels in the vaginal tracts of non-human mammals may prevent lactobacilli dominance (Mirmonsef et al., 2014). Furthermore, recent work suggests that, in addition to glycogen, the mammalian enzyme α-amylase must also be present in the vaginal tract to break down glycogen into a form usable by lactobacilli strains that cannot metabolize glycogen directly (Spear et al., 2014, 2015; Nasioudis et al., 2015). It is currently unknown whether α-amylase is present in the vaginal tract of mammals other than humans, but a lack of this enzyme in non-human mammals might also contribute to the absence of lactobacilli dominance in non-human mammals. A comparison of α-amylase gene copy number to vaginal pH may provide further evidence for the function of this enzyme in the vaginal tract. Additionally, quantification of glycogen and α-amylase levels in mammalian vaginal tracts, including humans, would enable researchers to identify species with “lactobacilli-friendly” vaginal tracts.

**Table 3:** **Concentration of glycogen in the vaginal tract across mammalian species**.

**Order**	**Species**	**Common name**	**Vaginal glycogen content**		**References**
			**Tissue (μg/100 mg of wet tissue)**	**Genital fluid (Glycogen:Protein [μg/μg])**	
**RODENTIA**					
	*Mesocricetus auratus*	Golden Hamster	182.78	–	Gregoire and Guinness, 1968
	*Mus musculus*	House Mouse	68.98	–	Balmain et al., 1956
	*Oryctolagus cuniculus*	European Rabbit	44.00	–	Gregoire and Hafs, 1971
	*Rattus norvegicus*	Brown Rat	35.56	–	Shukla et al., 1989
**PRIMATES**					
	*Homo sapiens*	Human (non-BV)	1395.75	–	Gregoire et al., 1971
			–	0.2	Mirmonsef et al., 2012
		Human (BV)	–	0.04	Mirmonsef et al., 2012
	*Macaca mulatta*	Rhesus Macaque	603.67	–	Gregoire and Parakkal, 1972
			–	0.004	Mirmonsef et al., 2012
	*Macaca nemestrina*	Pig-Tailed Macaque	–	<0.001	Mirmonsef et al., 2012

Assuming that human vaginal tracts do have higher levels of glycogen than other mammals, the next question is: why do humans exhibit such high levels of glycogen? From an evolutionary perspective, high glycogen may be the result of selection for a protective microbial community, as suggested by the disease risk and obstetric risk hypotheses. However, given the lack of evidence for these hypotheses, we suggest that glycogen abundance is a byproduct of some other aspect of human physiology or behavior. In particular, we propose a “diet hypothesis,” which centers on the high starch content of human diets. Humans ingest relatively large quantities of starch, facilitated by the origins of agriculture, cooking food, and our ability to efficiently break carbohydrates down with high levels of amylase in saliva (Englyst et al., 1992; Diamond, 2002; Perry et al., 2007; Carmody and Wrangham, 2009; Hardy et al., 2015). Because glycogen is the major storage molecule of glucose, high starch diets may have led to high levels of glycogen in the vaginal tract, which, in turn, might create a favorable environment for lactobacilli proliferation. Although the mechanism by which high starch diets might lead to high levels of glycogen in the vaginal tract is unknown, it is well established carbohydrate ingestion increases glycogen in the liver and skeletal muscle (McGarry et al., 1987; Jeukendrup, 2003). In addition, there is some evidence that differences in women's diets predict differences in glycogen levels in the vagina and BV risk. For instance in one study, having a BMI > 30 was linked to increased free glycogen in vaginal fluid, although this relationship was only marginally significant (Mirmonsef et al., 2014). Further, diet may play a role in vaginal microbial composition, particularly with regard to risk of BV (Neggers et al., 2007; Tohill et al., 2007; Thoma et al., 2011b). A priori, we might hypothesize that a shift to diets rich in starch transformed vaginal microbial communities (and pH), and that evidence of this history might be found in comparisons among women with different ancestries. For example, it has been shown that individuals from human lineages that consumed more starch (e.g., agrarian societies as opposed to hunter gatherers) are more likely to have more copies of salivary amylase genes and, in turn, produce more amylase (Perry et al., 2007). Interestingly, there is variation in vaginal pH and community composition among human populations (Ravel et al., 2011; MacIntyre et al., 2015). However, because this variation is typically matched to race rather than to genotype, it is difficult to know whether this variation is in line with our prediction or not. To date, only one study has investigated the affect of a high starch diet on vaginal glycogen levels in humans (Willson and Goforth, 1942). Although the researchers did not detect a change in glycogen after the implementation of an abnormally high carbohydrate diet, we do not believe this finding represents an adequate test of this hypothesis as subjects were postmenopausal and glycogen concentrations were not assessed quantitatively.

## Conclusions and future directions

Of the four hypotheses currently proposed to explain the unique nature of the human vaginal microbiome, our results provide little to no evidence for the reproductive phase, disease risk, and obstetric protection hypotheses. The fourth, common function hypothesis and our newly proposed diet-based hypothesis are as yet untested and represent two promising areas for future research. Overall, future work on the vaginal microbiome, particularly with regard to mechanistic and evolutionary explanations for human uniqueness, will require additional comparative data. Specifically, it would be useful to have more data on the vaginal microbiome of wild populations of mammals, especially from different stages of the ovarian cycle. It would also be useful to characterize vaginal microbiomes and vaginal pH in species of mammals with especially high obstetric risk, such as the hyena (Frank and Glickman, 1994), or similar patterns of socio-sexual behavior to humans, such as the bonobo or bottlenose dolphin (Dixson, 2012; Furuichi et al., 2014). Furthermore, there is an urgent need for more information on both glycogen and α-amylase content of the vaginal tract in both humans and other mammals. This type of data would be particularly useful for understanding the mechanistic origins of low vaginal pH in humans and exploring the relationship between the human vaginal tract and diet. To fully test the human diet hypothesis, however, both experimental and comparative work will be required, including the comparison of vaginal microbiota across human populations with differing diets, such as hunter-gatherers and agricultural societies.

## Author contributions

Study concept and design was a collaboration between all authors. Literature searches and data collection were carried out by EM and DB. All statistical analyses were performed by EM. The manuscript was written by EM and EA with input from DB and RD.

## Funding

This work was supported by the National Science Foundation (DGE-1313583 to EM, IOS-1053461 to EA, and MSP-1319293 to DB).

### Conflict of interest statement

The authors declare that the research was conducted in the absence of any commercial or financial relationships that could be construed as a potential conflict of interest.
