# Supplementary material for: Lactobacilli Dominance and Vaginal pH: Why Is the Human Vaginal Microbiome Unique?
Source: Front Microbiol. 2016 Dec 8;7:1936. doi: 10.3389/fmicb.2016.01936 (PMC5143676; doi:10.3389/fmicb.2016.01936)
Supplement: Supplementary file 3 [file Image1.PDF]

Supplementary Material

Lactobacilli Dominance and Vaginal pH: Why is the Human Vaginal Microbiome Unique?

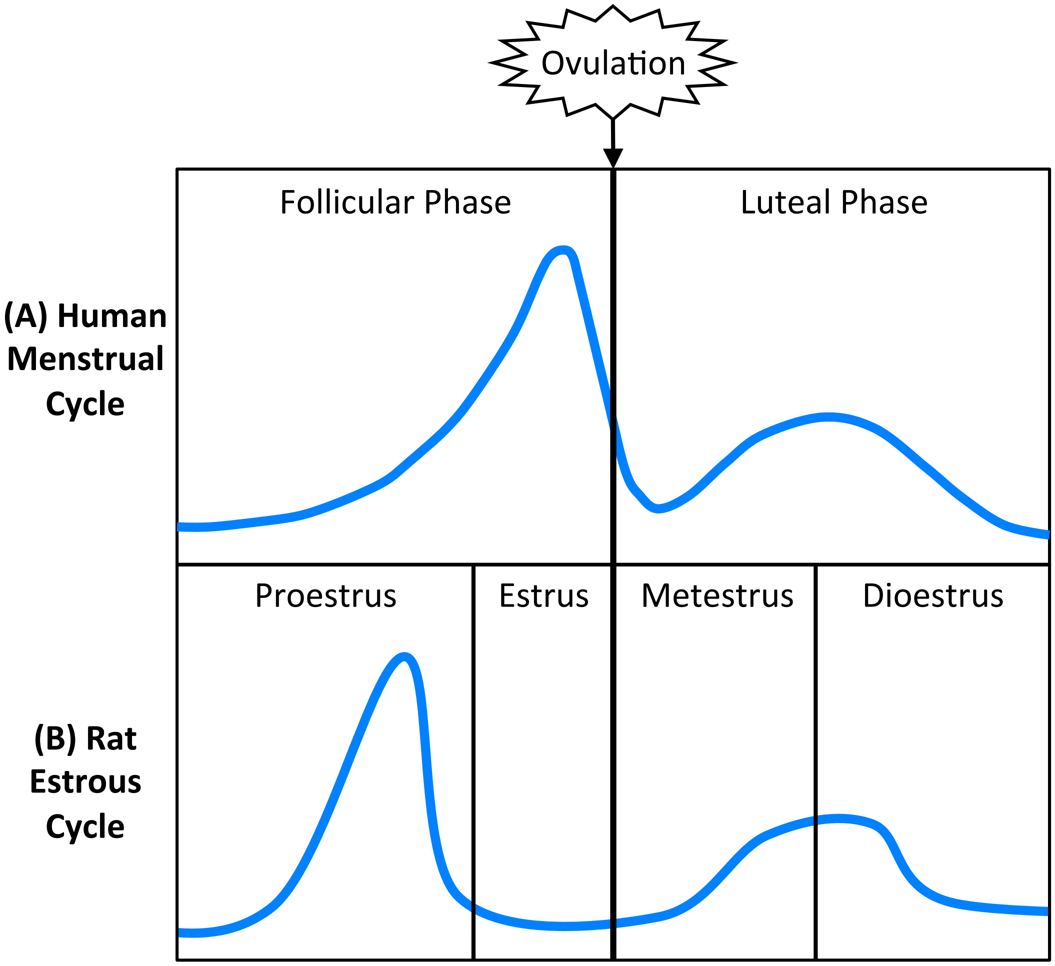

Supplementary Figure 1. Fluctuations in estrogen during the menstrual cycle versus the estrous cycle as represented by the human and rat. (**A**) The menstrual cycle is separated into two phases–the follicular phase and the luteal phase–by ovulation. In humans, estrogen concentrations peak during the follicular phase, before ovulation, and then decline to a low level at beginning of the follicular phase, when menstruation occurs. (**B**) The estrous cycle is divided into four parts, with proestrus and estrus collectively referred to as the follicular phase, and metestrus and dioestrus referred to as the luteal phase. In rats, estrogen levels are highest during proestrus and then drop to low levels at the end of metestrus and beginning of dioestrus.
